# Policies of voluntary services involved in public health emergencies in China: Evolution, evaluation, and expectation

**DOI:** 10.3389/fpubh.2022.946888

**Published:** 2022-08-18

**Authors:** Hongli Chen, Jing Wang, Xiaohong Yu, Cheng Li, Yue Zhao, Ying Xing, Xianwen Li

**Affiliations:** School of Nursing, Nanjing Medical University, Nanjing, China

**Keywords:** public health emergency, voluntary service, policy document, textual analysis, policy instrument

## Abstract

**Background:**

Public health emergencies have an immense effect on social stability, economic development, and human life. Volunteers played an indispensable role in health monitoring, assistance with daily life, and social network repairing. However, few policies analysis concentrated on the voluntary services involved in public health emergencies.

**Objectives:**

This study aims to analyze and summarize the advantages and flaws of the policy documents relating to voluntary services involved in public health emergencies, and put forward the enlightenment on policymaking and optimization.

**Methods:**

A three-dimensional analysis framework of “Policy instruments—Participants of voluntary services—Stages of voluntary services” was designed. Policy documents at the national level were retrieved from the official websites of the State Council of the People's Republic of China and its departments as well as the PKULAW Database. NVivo software was applied to analyze the contents of the included policy documents. Gephi software was adopted to conduct a visualized atlas analysis of the cooperative network among policymaking departments.

**Results:**

A total of 77 policy documents were included, and most were published in 2020 (*n* = 40). The Ministry of Civil Affairs (*n* = 19) and the National Health Commission (*n* = 18) issued more documents than the other departments. They cooperated more extensively with other departments. In policy documents, supply-side policy instruments were utilized the most (65.4%), followed by demand-side (23.9%). Voluntary organizations in the form of ambiguous sense were most mentioned as service participants (*n* = 73). In the stages of service delivery, service content mainly involved the prevention and control of public health emergencies (27, 18.9%) and psychological counseling (26, 18.2%).

**Conclusion:**

Time distribution of policy documents featured “incubation period—outbreak and continuous evolution period—elimination recovery period.” Joint issuing became the dominant form. The internal structure of policy instruments was unbalanced with different priorities, and the overall structure is expected to be optimized to promote voluntary organization management, reinforce external resources, and close the gap between policymaking and policy implementation. Volunteers' competence and voluntary organizations' system needs to be improved, and the contents of voluntary services should be enriched for the preparedness for future public health emergency.

## Introduction

Public health emergencies have an immense effect on social stability and economic development (1), with various detrimental impacts on human life and physical and psychological health as we know it (2, 3). Since the beginning of the twenty-first century, six outbreaks of infectious diseases have been declared as Public Health Emergency of International Concern (PHEIC) by the World Health Organization (WHO), starting with the Influenza A (H1NI) pandemic in 2009, and the ongoing global pandemic of coronavirus disease 2019 (COVID-19) first identified in December 2019, which is the worst pandemic of this century in terms of incidence and number of deaths (4). Although the virus shares about 86% homology with the virus of Severe Acute Respiratory Syndrome (SARS) that outbroke in China in 2003, and shares similar clinical manifestations with SARS (5), COVID-19 is much worse. It has been characterized by fast transmission, widespread, and difficult prevention and control (6). Subsequently, nearly 200 cases of unexplained hepatitis have been reported in children globally and 17 children required a liver transplant according to a WHO Alert on April 2022 (7). Much attention is being given to the cause and response to the public health emergencies in global countries.

During the past two decades, social volunteers have been a vital role in the prevention and control of public health emergencies. Since the initial outbreak of COVID-19, China has attached great importance to it, and comprehensively strengthened the unified organization and coordination of the entirety of society to block the COVID-19 transmission, which has effectively prevented a far worse situation (8, 9). Volunteers, as social forces, have played an indispensable role in the popularization of medical knowledge, health monitoring, assistance with daily life, and psychological counseling during the outbreak and continuous evolution period of the pandemic in China, and played a key role in social network repairing, that is, grief counseling during the elimination recovery period (10, 11). Even conservatively estimated, more than 300 million volunteers and 12,000 volunteer organizations participated in the pandemic prevention and control (12). Meanwhile, various government departments of the State Council have issued a series of pandemic prevention and control policies, providing the theoretical basis for promoting the participation of volunteers in services and guiding their implementation (13, 14).

However, the analysis of policy documents in the field of public health emergencies such as COVID-19 only focused on the characteristics from a wide perspective, while few systematic policy documents analysis concentrated on the participation of voluntary organizations or volunteers in the prevention and control of public health emergencies (15). This paper aims to generate fresh insight into this research gap. Based on the three-dimensional analysis framework designed by the research group, we analyzed the policy documents in this field under the background of public health emergencies in China and summarized the existing policy characteristics of evolution, types, policymaking departments, and advantages, which aimed to support and give implications in policymaking to the other countries in which COVID-19 has been reported. As well, the flaws were evaluated to put forward the policy optimization suggestions, through which we expect to lay a theoretical and policy foundation for the institutional and systematical construction of voluntary organizations participating in public health emergencies.

## Materials and methods

### Data collection

The policy documents at the national level on the official websites issued by The State Council of The People's Republic of China and its constituent departments, encompassing the National Health Commission, Ministry of Civil Affairs, National Development and Reform Commission, Ministry of Industry and Information Technology, and other relevant commissions or ministries, were retrieved. All the policy documents are open to access on the official websites. As well, to comprehensively obtain the policy documents, we searched for the PKULAW Database for supplementation in preventing missing retrieving documents on official websites. It is the largest Chinese policy full-text database compiling public policy documents promulgated in China since 1949. The full text of documents can be accessible after purchasing the database. The search strategy was “(volunteer OR voluntary organization) AND (public health emergency OR pandemic).” The inclusion criteria for selecting policy documents were as follow: (a) The participation of voluntary organizations in the prevention and control of public health emergencies should be explicitly mentioned in the policy content; (b) The types of policy document included notifications, schemes, implementation plans, laws and regulations, and other documents reflecting government policies; (c) Considering the SARS epidemic occurred in 2003 as the first public health emergency in the People's Republic of China, policy documents issued at the national level since the twenty-first century (From 1 January 2000 to 31 March 2022) were selected. We excluded studies by the following criteria: (a) News reports, conference speeches, work reports, and interpretation of relevant policy documents; (b) Policy documents that duplicate those already included in the policy document pool. The retrieval of policy documents was carried out in April 2022. The included policy documents concerning the participation of voluntary organizations in the prevention and control of public health emergencies are shown in Supplementary Table 1, with a total of 77 documents.

### Research methods

**Figure 1:**
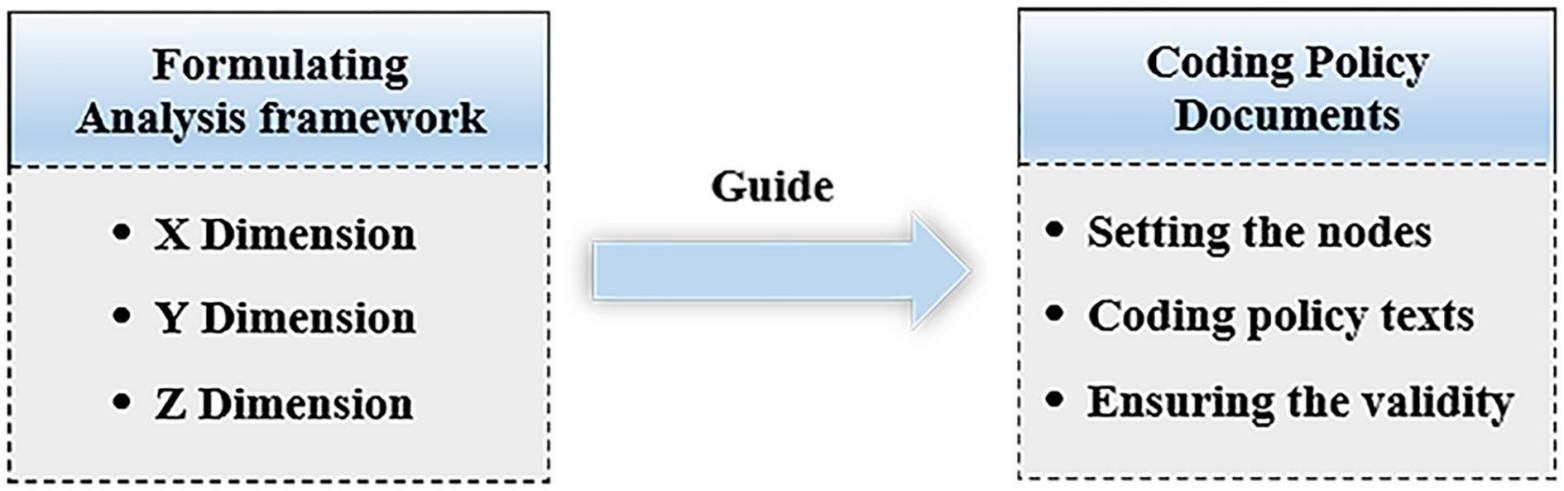
The analysis process for policy documents.

The study adopted a policy document textual analysis method, which is a research method for rigorously and systematically analyzing the contents of policy documents' full text outlined by Cardno (16). It includes two different ways: inductively and deductively. We used the latter. In the deductive approach, very often a model or theory established is the basis for textual analysis of content. Thus, two key steps of the deductive content analysis were concluded, containing: (a) setting an analysis framework; and (b) document coding including setting the nodes, coding policy texts, and ensuring the validity. An overview of what each of these steps entails is shown in Figure 1.

#### Analysis framework formulating

**Figure 2:**
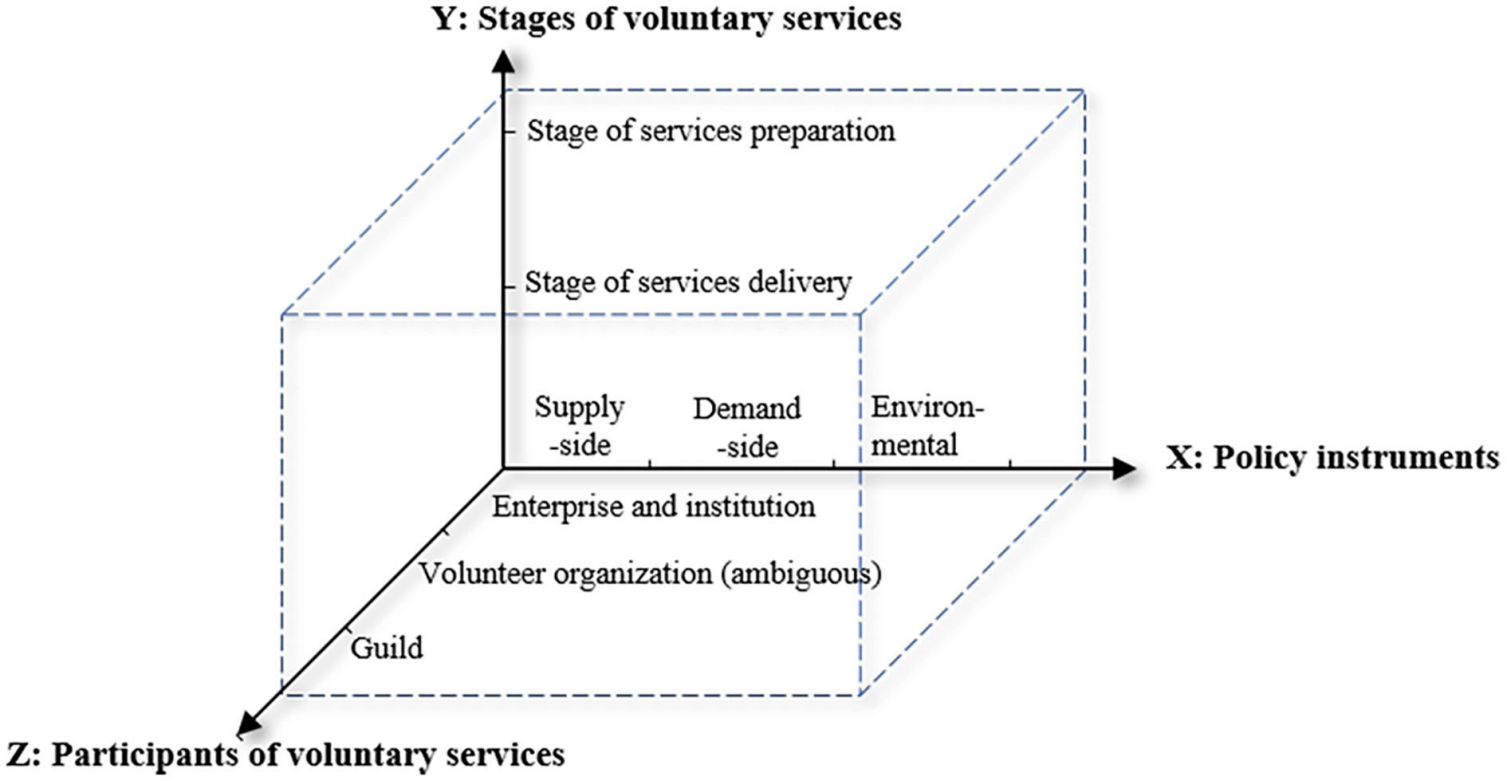
Analysis framework regarding voluntary services in public health emergencies.

Rothwell and Zegveld's (17) classical taxonomy of policy instruments simplifies the complex policy system. It weakened the compulsion of government and highlighted the role of the government departments in promoting the development of the environment in a certain field. The significant impact of environmental shaping, and supply and demand improvement were also emphasized in this classification of policy instruments, which is in line with the development needs of voluntary services. In addition, under the background of the growth of public demand and the shortage of public service, social governance system based on multi-center governance theory emphasized the diversity of social forces participation, also the development rules and characteristics of services should be considered when the policymakers make policies (18). Therefore, in the context of voluntary organizations participating in public health emergencies, it is necessary to establish an analysis framework regarding the above dimensions to formulate a research perspective for the interpretation of existing policy documents. The “Policy instruments—Participants of voluntary services—Stages of voluntary services” analysis framework was set, as shown in Figure 2.

X Dimension: policy instruments. Rothwell and Zegveld's classical classification of policy instruments, including supply-side, demand-side, and environmental policy instruments, was adopted to form the X dimension of the policy analysis framework. The supply-side policy instruments are the government departments directly providing resources to support and promote voluntary organizations to participate in the services of public health emergencies. The demand-side policy instruments reflect the government departments' pulling role in encouraging voluntary organizations to participate in services. The environmental policy instruments are the government departments creating a favorable macro policy environment for the sustainable development of voluntary organizations. To better understand the above policy instruments and facilitate the extraction and coding of policy documents, the specific connotation of each instrument was set in conjunction with the features of the development of voluntary services in China. The connotation of each policy instrument is shown in Table 1.

**Table 1:** The description of policy instruments.

**Policy type**	**Instrument name**	**Description**
Environmental policy instruments	Goal programming	Making an overall target and plan for voluntary service organizations to participate in the prevention and control of public health emergencies.
	Standard and regulation	Formulating a series of standards to regulate the responsibilities and services implementation of voluntary service organizations.
	Performance appraisal	Setting up a social supervision platform, improving the third-party evaluation mechanism, and encouraging local governments to refine services evaluation indicators based on the actual situation of voluntary organizations participating in public health emergencies.
	Strategic measures	Refining specific and feasible strategies for the macro goal programming.
Supply-side policy instruments	Education and Training	Providing education and training of volunteers needed by social voluntary organizations to strengthen the capacity of the teams participating in public health emergencies.
	Financial support	Providing financial support for voluntary organizations.
	Organizing and guiding	Leading and strengthening work guidance over the participation of voluntary organizations in public health emergencies.
	Infrastructure	Integrating the resources of existing communities, medical institutions, and social organizations to provide the basic guarantee for volunteers participating in the prevention and control of public health emergencies.
	Science and technology support	Promoting the improvement of the management platform for voluntary organizations. Strengthening the interconnection of the information management systems between voluntary service organizations and professional institutions. Supporting the design of emergency response and prevention services by expert teams.
Demand-side policy instrument	Government purchase	Government purchasing the prevention and control services of public health emergencies from third-party organizations, to stimulate the participation of various social organizations, such as health industry associations, universities, and public welfare organizations.
	Encouragement and mobilization	Encouraging the use of new media communication technologies, such as Television, WeChat, and Weibo, to promote the publicity of voluntary services, and promote the participation of different subjects in public health emergencies.

Y Dimension: stages of voluntary services. Reviewing the policy analysis of voluntary services through literature, we found that the law of voluntary service development and the characteristics of activities should be taken into account when policymakers make policies (19). Therefore, the study divided the process of voluntary service activities development into two stages: service preparation and service delivery. The stages of service preparation are the preliminary preparation of voluntary services, encompassing preparation of infrastructure, goal setting and system formulating, volunteer registration, recruitment and training, and funding preparation. The stages of service delivery contain the stages of scheme establishing and implementation of voluntary service activities.

Z Dimension: participants of voluntary services. Volunteer, as a basic unit to perform or offer services voluntarily contributing to social development, is an important power in participating in the services of prevention and control of public health emergencies. We took this dimension into consideration. Any social force or anyone can be a volunteer, containing charitable organizations, enterprises and institutions, non-governmental organizations, guilds, and so on (20).

#### Policy documents coding and analysis methods

At the very heart of the textual analysis of the contents is document coding. First, we set the nodes according to the above three dimensions of the analysis framework. And then, the NVivo 12 software was used to code the content of 77 policy texts included in the study. Combined with the application of Excel software, descriptive analysis was conducted on the types of policy instruments the policy documents used, the participants of voluntary services, the stages of voluntary services, as well as the general characteristics including department, time, and type of policy documents. In addition, we regarded the policy documents issued by multiple government departments jointly as the cooperation between the subjects and determined their cooperation matrix of them. Gephi 0.9.3 was adopted to conduct a visualized atlas analysis of the cooperation network among different departments. To ensure the consistency and reliability of the coding, two researchers independently screened the policy documents and extracted the data. Any disagreement was resolved through discussion, and a third researcher arbitrated the disagreement.

## Results

### General characteristics of the policy documents

#### The evolution of policy documents

**Figure 3:**
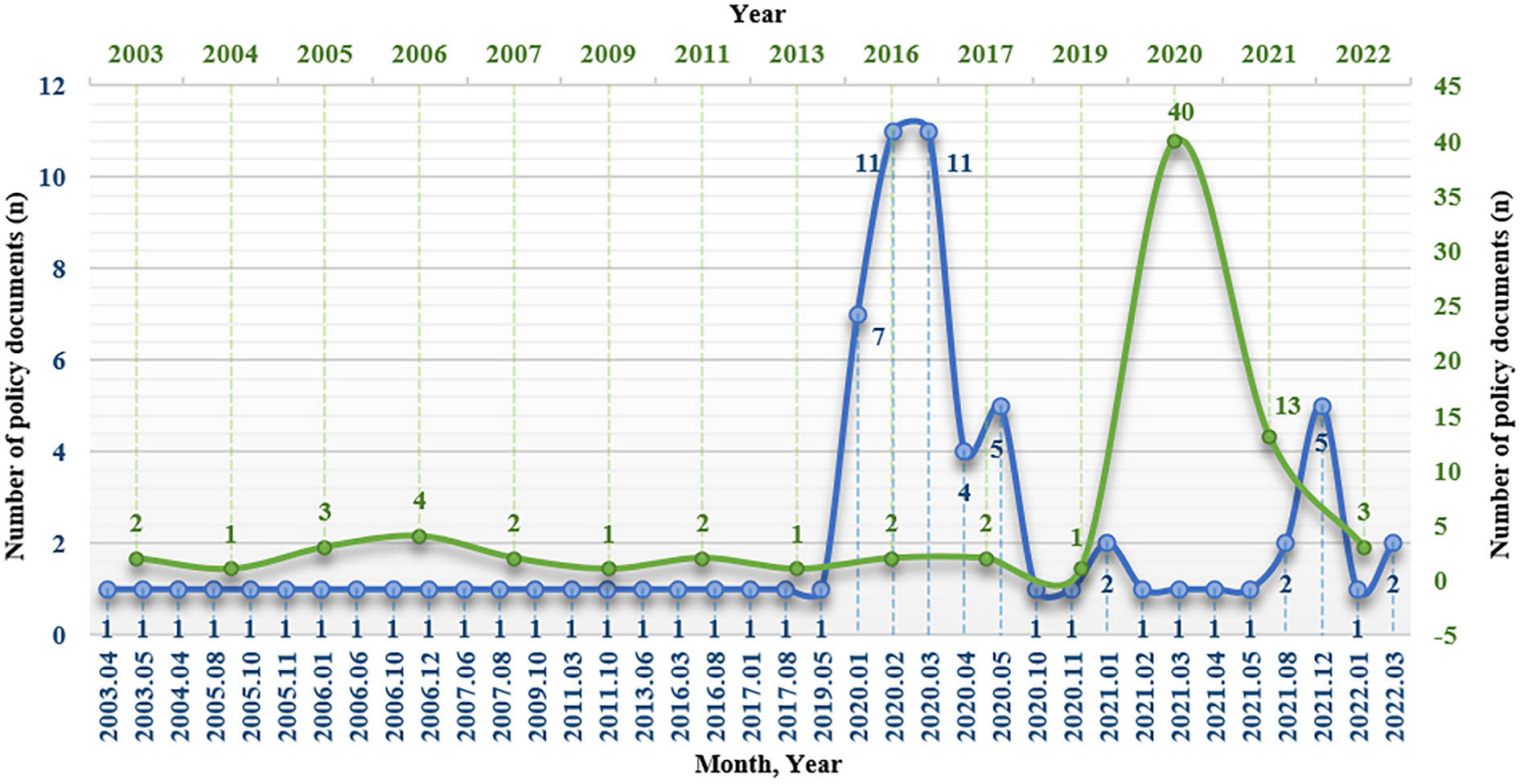
Time distribution of included policy documents.

As shown in Figure 3, from 2003 to 2019, the number of policy documents concerning the participation of voluntary organizations in the prevention and control of public health emergencies at the national level fluctuated steadily from 0 to 4 per year. In 2020, the number of policy documents in this field reached the maximum of 40. The fluctuation features of the number of policy documents in each month from 2020 to 2022 were similar to the characteristics of the phases of the COVID-19 outbreak and regular prevention and control in China, presenting that the number of policy documents suddenly increased from 7 to 11 from January to March, and then dynamically fluctuated from 0 to 5.

#### Policymaking departments and types of policy documents

**Figure 4:**
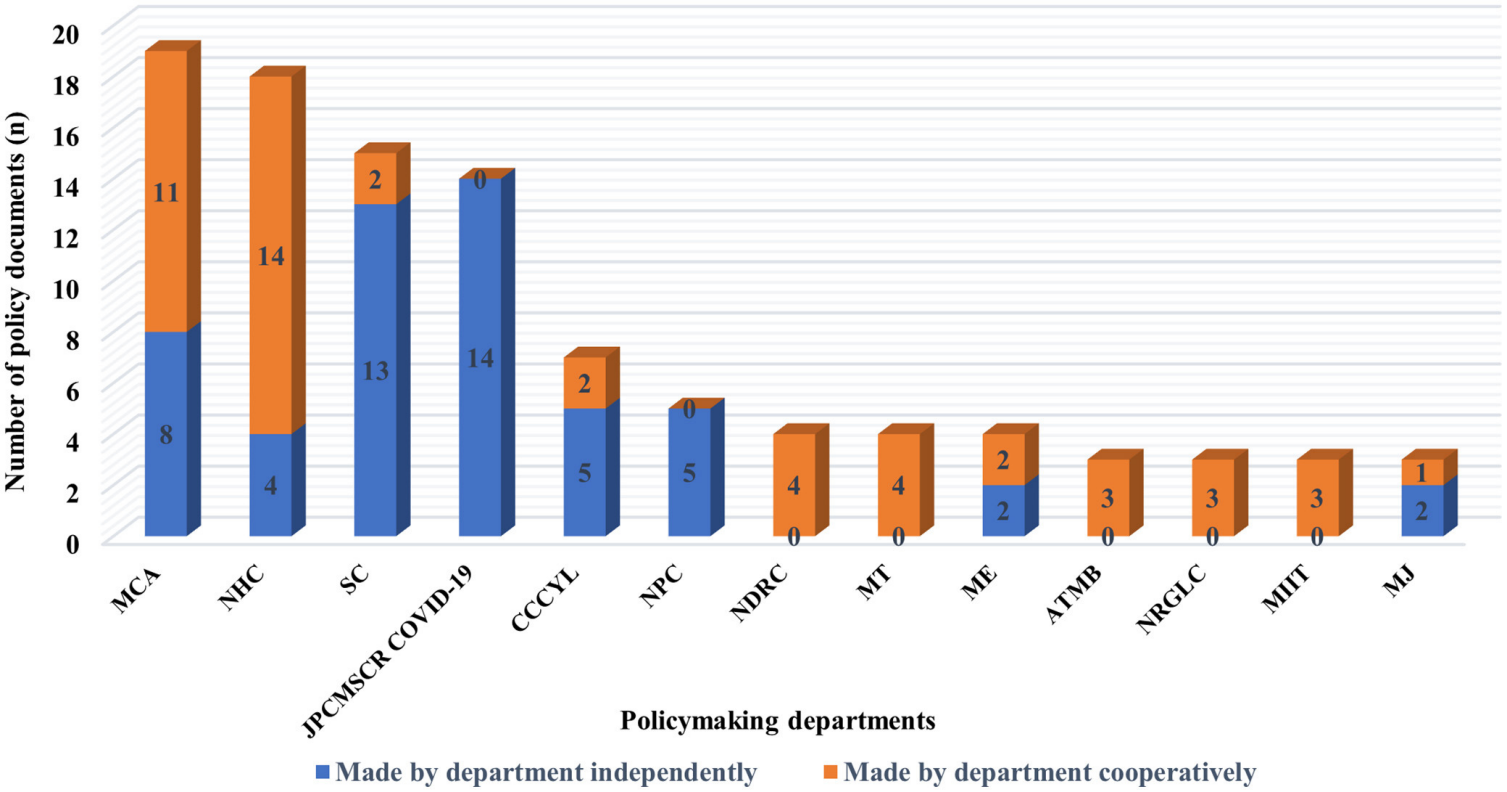
Number of policy documents issued by departments (top 13). MCA, Ministry of Civil Affairs; NHC, National Health Commission; SC, State Council; JPCMSCR COVID-19, Joint Prevention and Control Mechanism of the State Council in Response to COVID-19; CCCYL, Central Committee of the Communist Youth League; NPC, National People's Congress; NDRC, National Development and Reform Commission; MT, Ministry of Transport; ME, Ministry of Education; ATMB, Air Traffic Management Bureau; NRGLC, National Railway Group Limited Company; MIIT, Ministry of Industry and Information Technology; MJ, Ministry of Justice.

According to the current name of each government department, the number of departments that issued the policy documents was counted. Ministry of Civil Affairs (*n* = 19), National Health Commission (*n* = 18), State Council (*n* = 15), and Joint Prevention and Control Mechanism of the State Council in Response to COVID-19 (*n* = 14) released more policy documents than the other departments, including independent and cooperative release (Figure 4).

**Figure 5:**
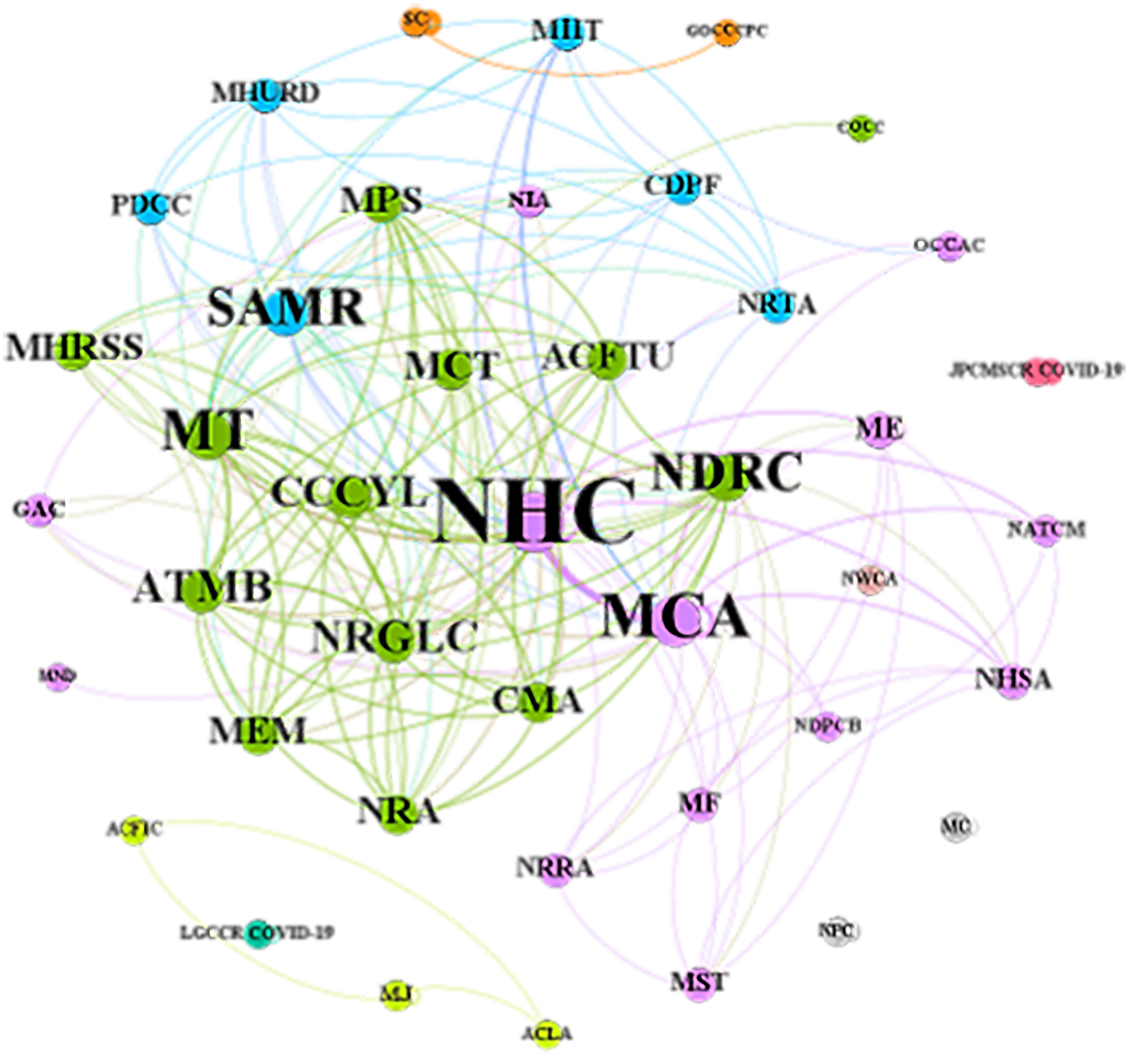
The visualization map of policymaking departments' cooperative network. MCA, Ministry of Civil Affairs; NHC, National Health Commission; SC, State Council; JPCMSCR COVID-19, Joint Prevention and Control Mechanism of the State Council in Response to COVID-19; CCCYL, Central Committee of the Communist Youth League; NPC, National People's Congress; NDRC, National Development and Reform Commission; MT, Ministry of Transport; ME, Ministry of Education; ATMB, Air Traffic Management Bureau; NRGLC, National Railway Group Limited Company; MIIT, Ministry of Industry and Information Technology; MJ, Ministry of Justice; SAMR, State Administration for Market Regulation; LGCCR COVID-19, Leading Group of Central Committee on Response to COVID-19; MEM, Ministry of Emergency Management; GOCCCPC, General Office of Central Committee of the Communist Party of China; MCT, Ministry of Culture and Tourism; NATCM, National Administration of Traditional Chinese Medicine; MPS, Ministry of Public Security; CMA, China Meteorological Administration; NRA, National Railway Administration; ACFTU, All-China Federation of Trade Unions; NHSA, National Healthcare Security Administration; MC, Ministry of Commerce; CDPF, China Disabled Persons' Federation; NWCA, National Working Commission on Aging; NDPCB, National Disease Prevention and Control Bureau; COCC, Civilization Office of Central Committee; MHRSS, Ministry of Human Resources and Social Security; MND, Ministry of National Defense; PDCC, Propaganda Department of Central Committee; MHURD, Ministry of Housing and Urban-Rural Development; NRTA, National Radio and Television Administration; GAC, General Administration of Customs; NIA, National Immigration Administration; ACFIC, All-China Federation of Industry and Commerce; ACLA, All-China Lawyers Association; OCCAC, Office of the Central Cyberspace Affairs Commission; MST, Ministry of Science and Technology; MF, Ministry of Finance; NRRA, National Rural Revitalization Administration.

The cooperation network among various government departments is shown in Figure 5, with 42 subjects constituting 189 cooperation edges. A larger font size of name and node indicates that the government department has more cooperation with others. The thicker the edge, the closer the cooperative network between two government departments. The National Health Commission (NHC), Ministry of Civil Affairs (MCA), National Development and Reform Commission (NDRC), Ministry of Transport (MT), and State Administration for Market Regulation (SAMR) cooperated more extensively with other departments, while the Joint Prevention and Control Mechanism of the State Council in Response to COVID-19 had no cooperation with other departments.

The classifications of policy documents can be divided into seven types: notification (35, 45.5%), scheme (11, 14.3%), plan (9, 11.7%), suggestion (9, 11.7%), guideline (8, 10.4%), law (3, 3.9%), and regulation (1, 2.6%).

### Textual analysis of policy documents' contents based on analysis framework

#### Policy instruments formulated by the government departments

Among the 77 policy documents, supply-side policy instruments were the most used, accounting for 65.4%. The government departments focused mostly on organizing and guiding (56, 53.8%), followed by education and training (17, 16.3%) and science and technology support (15, 14.4%). The demand-side policy instruments were accounting for 23.9%. The frequency of encouragement and mobilization was the highest (34, 89.5%) among demand-side policy instruments. Under public health emergencies, systematic and institutionalized government purchase was seldom used (4, 10.5%). Environmental policy instruments were involved the least, accounting for only 10.7%, and among which, goal programming (6, 35.3%) and performance appraisal (5, 29.4%) were the most utilized by the government departments (Table 2).

**Table 2:** Coded policy documents of policy instruments.

**Policy type**	**Instrument name**	**Number of coded**	**Proportion**	**Total**	**Proportion**
		**policy documents (n)**			
Environmental policy instruments	Goal programming	6	35.3%	17	10.7%
	Standard and regulation	3	17.6%		
	Performance appraisal	5	29.4%		
	Strategic measures	3	17.6%		
Supply-side policy instruments	Education and Training	17	16.3%	104	65.4%
	Financial support	7	6.7%		
	Organizing and guiding	56	53.8%		
	Infrastructure	9	8.7%		
	Science and technology support	15	14.4%		
Demand-side policy instruments	Government purchase	4	10.5%	38	23.9%
	Encouragement and mobilization	34	89.5%		

#### Nature of different participants and stages of voluntary services

**Figure 6:**
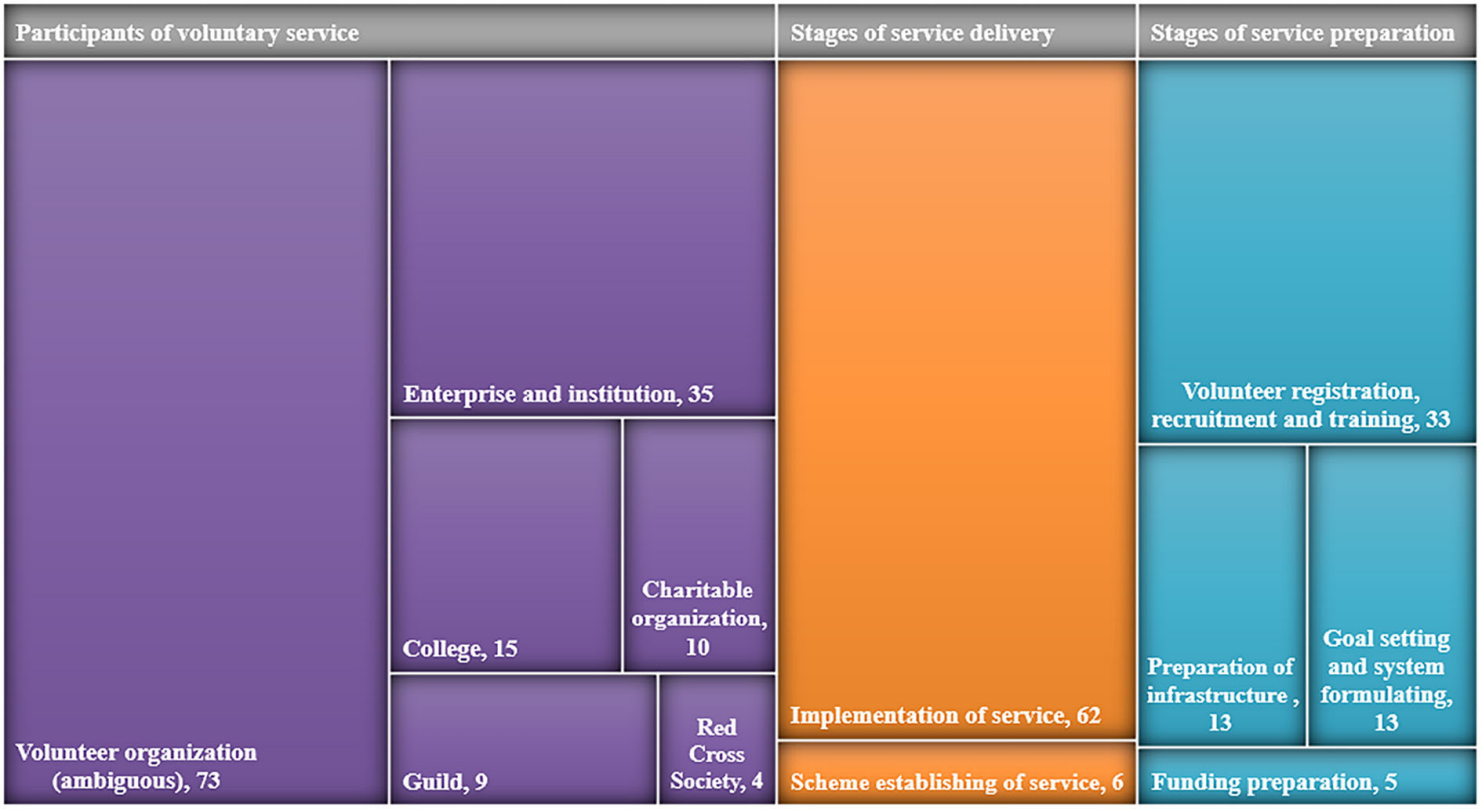
Treemap of stages and participants of voluntary services (n: number of coded policy documents).

It can be seen from Figure 6 the results of different participants and stages of social voluntary services in public health emergencies. Voluntary organizations (ambiguous) (73) were the most mentioned among the participants of voluntary services, while the demand for medical institutions and enterprises (35), colleges (15), guilds (9), and other professionals to participate in the voluntary service of public health emergencies was presented as well. In the comparison of the service stages, the number of coded policy documents in the service delivery stages was similar to that in the service preparation stages (*n* = 68 and *n* = 64, respectively). In the stages of service preparation, volunteer registration, recruitment, and training were the most, with the number of coded policy documents being 33. The implementation of service was mentioned the most in the stages of service delivery, with the number of coded policy documents being 62. The specific produced service contents mainly consisted of public health emergency prevention and control (27, 18.9%), psychological counseling (26, 18.2%), popularization of medical knowledge and training (23, 16.1%), assistance with daily life (21, 14.7%), supply of living and preventing material (13, 9.1%), health service and monitoring (11, 7.7%), and so on (Table 3).

**Table 3:** Contents of voluntary services of public health emergencies.

**Services**	**Number of coded**	**Proportion**
	**policy documents**	**(%)**
	**(*n*)**	
Policy interpretation	4	2.8
Health service and monitoring	11	7.7
Social network repairing	8	5.6
Supply of living and preventing material	13	9.1
Assistance with daily life	21	14.7
Public health emergency prevention and control	27	18.9
Popularization of medical knowledge and training	23	16.1
Psychological counseling	26	18.2
Referral service	4	2.8
Others (grief counseling, legal counseling, palliative and hospice care, and reproduction guiding)	6	4.2

#### Two-dimensional cross analysis between policy instruments and service stages

As shown in Table 4, among environmental policy instruments, goal programming and strategic measures instruments were both used in stages of service preparation and service delivery, while standard and regulation instrument was often utilized before the implementation of the services. The use of performance appraisal was mainly for the implementation of services. The education and training, financial support, and infrastructure construction in supply-side policy instruments mainly focused on the stages of service preparation, which matches the contents in this stage. Science and technology support was mentioned both in the stages of service preparation and service delivery. Encouragement and mobilization instrument was more often applied by the government departments to push the social forces to provide services voluntarily under public health emergencies.

**Table 4:** Two-dimensional analysis of coded policy documents between policy instruments and service stages.

**Type of instruments**	**Instruments**	**Stages of service preparation**		**Stages of service delivery**	
		**Number of coded**	**Total (*n*/%)**	**Number of coded**	**Total (*n*/%)**
		**policy documents (*n*/%)**		**policy documents (*n*/%)**	
Environmental policy instruments	Goal programming	6 (50%)	12 (16.7%)	6 (42.9%)	14 (12.8%)
	Standard and regulation	3 (25%)		0 (0.0%)	
	Performance appraisal	0 (0.0%)		5 (35.7%)	
	Strategic measures	3 (25%)		3 (21.4%)	
Supply-side policy instruments	Education and Training	16 (29.1%)	55 (76.4%)	2 (3.2%)	62 (56.9%)
	Financial support	7 (12.7%)		2 (3.2%)	
	Organizing and guiding	13 (23.6%)		49 (79.0%)	
	Infrastructure	10 (18.2%)		1 (1.6%)	
	Science and technology support	9 (16.4%)		8 (12.9%)	
Demand-side policy instruments	Government purchase	0 (0.0%)	5 (6.9%)	5 (15.2%)	33 (30.3%)
	Encouragement and mobilization	5 (100%)		28 (84.8%)	

## Discussion

### Discussion on general characteristics of policy documents

#### Time evolution characteristics of policy documents: Incubation period, outbreak and continuous evolution period, elimination recovery period

The temporal evolution of the policies related to voluntary organizations participating in public health emergencies presented similar evolutionary characteristics to the occurrence phases of public health emergencies in China, particularly during the period of the COVID-19 pandemic. Based on the theory of crisis lifecycle in conjunction with reality, we divided it into three stages: incubation period, outbreak and continuous evolution period, and elimination recovery period (21). The outbreak of SARS in China in 2003 was the prelude to mobilizing social volunteers to participate in the prevention and control of public health emergencies at the policy level. The Central Committee of the Communist Youth League, as the leading force, called on youth league members to actively participate in the prevention and control volunteer work in both urban and rural areas (22, 23). From 2003 to 2019, the number of policy documents related to this field issued at the national level fluctuated steadily from 0 to 4 per year. On the one hand, it related to the early development of voluntary service in China, and the service system was not yet sound during this period. On the other hand, there was no PHEIC being pandemic in China during this period. Therefore, under the background of static management form of prevention of public health emergencies, the formulation of policies in this field was also in a state of stable development. It can be described as the incubation period, during which we could make full preparation for public health emergencies.

In January 2020, COVID-19 broke out in China and continued to evolve from February to April. During the outbreak and continuous evolution period, China adopted the closure and community grid management mode to effectively block the transmission and avoid further expansion of the COVID-19 pandemic. In addition to the necessary medical staff and community workers participating in the prevention and control of pandemic, volunteers, as the active and altruistic human resources, played a significant role in daily necessities and mask delivery, and health monitoring. As well, a growing number of policy documents point out the essentiality of mobilizing and guiding volunteers to actively participate in the relevant work. With the announcement that the Wuhan lockdown lifted in April 2020 and the easing of the national pandemic situation, the government adhered to the general strategy of “foreign defense import, internal defense rebound” and the general policy of “dynamic clearing.” China has entered the period of regular pandemic prevention and control (24), with the number of policies related to voluntary organizations participating in COVID-19 prevention and control presenting dynamic and stable fluctuations. Now, it is in the elimination and recovery period.

#### Coordinated mode of policymaking departments and comprehensive application of document types

The cooperative network of policymaking departments presented a multi-body coordinative pattern, dominated by the National Health Committee, and with the Ministry of Civil Affairs, the National Development and Reform Commission, the State Administration for Market Regulation, and the Ministry of Transport as the local core. The National Health Commission and the Ministry of Civil Affairs cooperated most closely with each other, whose responsibilities matched best with the basic demands of humans' daily life and health, on which public health emergencies may have various detrimental impacts. Above two departments have played a leading and coordinating role in organizing and mobilizing volunteers to participate in pandemic prevention and control. Based on the analysis of the number of policy documents, the cooperative relationship between the National Development and Reform Commission, the Ministry of Transport, and other departments were extensive, while the depth of cooperation was lacking. The General Office of the State Council and the Joint Prevention and Control Mechanism of the State Council in response to COVID-19 also issued more policy documents in this field than the others. However, there was no cooperation or less cooperation with other departments. It should be noted that we could not accurately determine the participating departments. Often, multiple departments managed by them may be involved in policy formulation and release, so the current analysis was not able to clarify their cooperative relationship. Financial support is vital for the sustainable output of voluntary services. A previous study of policy analysis regarding response to public health emergencies has suggested that the Ministry of Finance cooperated in depth and extensively with other policymaking departments (16). However, the results of this study demonstrated that it is urgent to further develop cooperation in policymaking between the Ministry of Finance and other departments, and strengthen the financial support for voluntary service development under the background of public health emergencies.

The policy documents included in this study were mainly notifications, opinions, and plans, which were for instructing and assigning work, meanwhile still involved in schemes, guidelines, and regulations that can guide the implementation of practical work. During the SARS outbreak in 2003, the unified command of public health emergencies rescue system and legislation began to be established, marking the entry of the rule of law stage. *The Regulations on Emergency Response to Public Health Emergencies* and *National Overall Emergency Response Scheme for Public Health Emergencies* were released. The “China model” and “China experience” developed during the COVID-19 pandemic have brought the national public health emergency response system to a new level. Meanwhile, voluntary services have also been improved gradually during these stages (25). *The Regulations on Voluntary Service* and *Guidelines on Voluntary Organizations and Volunteers Participating in Pandemic Prevention and Control* were issued successively, which stipulated the participation of voluntary organizations in the prevention and control of public health emergencies. It reflects the formation process of continuous systematization and institutionalization of specific strategies during practice. But at the same time, the regulations' directivity on emergency voluntary services is still relatively limited and sketchy, leading hard to combine the public health emergency response and voluntary services well. Also, the specialized legal system for emergency volunteers and voluntary organizations management is still imperfect. The legalization and institutionalization guarantee to remain strengthened to guide and promote voluntary services of orderly participation together.

### Discussion on content characteristics of policy documents based on analysis framework

#### The internal structure of policy instruments is unbalanced and expected to be optimized

In general, the policies of volunteers participating in the prevention and control of public health emergencies in China have taken into account the comprehensive application of supply-side, demand-side, and environmental instruments. However, the utilization rate of policy instruments was different, and the internal structure was relatively unbalanced, with different emphases focused. The supply-side policy instrument was the most used, in which the proportion of organizing and guiding instruments was the highest. From the outbreak to the continuous evolution stage of COVID-19, volunteers were led by the unified organization of the departments of the Chinese government or the local governments in China. A governance model of “government-society” coordination has been formed, in which state authority and social participation are embedded in each other. In addition, in this context, the “Internet plus Service” supported by science and technology has aroused a strong mobilization force in a short time, which has changed the network from disorganization to self-organization, providing a vital foundation for the management and implementation of voluntary services. A study regarding volunteer corps during the COVID-19 pandemic indicated that the flexibility of remote opportunities allowed volunteers' involvement in services. They may otherwise have been unable to participate due to personal or financial constraints (26). The government departments advocated making full use of the information platform to achieve the aim of systematic management of volunteer registration and recruitment in the service preparation stage. In the service delivery stage, the Internet platform could be applied to provide services online, so as to reduce the risk of carrying out voluntary service activities of public health emergencies in offline form. The government departments also put forward certain requirements on volunteer education and training, financial support, and infrastructure construction which were mainly utilized in the service preparation stage and matched the contents of the service preparation. What needs to be noted is that other types supply-side instruments were all used less than the organizing and guiding. From the perspective of the preparation process of voluntary services, there are still some management problems such as temporary recruitment of volunteers, lack of systemic education and training, weak security for volunteers, and insufficient funding, which is similar to the viewpoints proposed by Wei et al. in China (27). Also, in Spain, Gómez-Durán et al. investigated volunteering experience perceptions of healthcare students during the COVID-19 pandemic, some of them reported similar problems in appropriate specific-task education before starting, supervision, and protective equipment (28). It is necessary to improve the unbalance in the formulation of supply-side policy and promote the optimization of voluntary organization management, containing education, training, financial support, etc., which are the prerequisite for volunteers to actively participate in services.

Among the demand-side policy instruments, the number of encouragement and mobilization instrument was the most. During the COVID-19 pandemic, the social pandemic management model had requirements of spontaneous, active, and voluntary human resources. The motivation to participate in pandemic prevention and control voluntary services was not only from internal but also from external forces. It was necessary to change their values, living habits, attitudes, and expectations through various forms of propaganda, making them produce a continuous expected behavior of obeying the unified national instruction (27). During the outbreak and continuous evolution period, systematic and institutionalized government purchase was seldom adopted, which may be more suitable for the period of regular management of public health emergencies. However, during the outbreak of public health emergencies, how to realize the participation of voluntary organizations from the form of “internal push, external pull” to systematized and institutionalized participation under the unified deployment and scheduling of the government, is not only the requirement in the regular prevention and control but also the preparedness for the next public health emergencies. First, it needs to solve the existing problems in the supply-side policy, so as to provide strong external resource support for voluntary organizations to participate in the prevention and control of public health emergencies. Second, government purchase, which is seldom used in this field, may also be a useful exploration.

The formulation of environmental policy instruments also needed to be improved. The number of goal programming instruments was the most related to the fact that most documents are issued in the form of notifications, plans, and suggestions, which only can provide practical work with some general direction. Although the existing policy documents also contain schemes, the directivity of voluntary participation in the prevention and control of public health emergencies is weak, and the establishment of standards, norms, and strategic measures still need to be updated and optimized in the process of practice which could provide theoretical guidance for the practical voluntary services. Additionally, it is necessary to improve the evaluation index system for the voluntary services in the prevention and control of public health emergencies and to clarify the subjects of supervision and evaluation. As well, we can make full use of the existing voluntary service online management platform to achieve the tracking evaluation of the services. Through the above improvement of environmental policy, the gap between policymaking and policy implementation can be closed to put supply-side policy and demand-side policy into practice.

#### The competence of volunteers should be improved and the contents of services should be enriched

The voluntary services in public health emergencies require the joint efforts of various parties. Medical institutions, enterprises, universities, and other professionals should be encouraged to provide voluntary services actively, or they could provide professional guidance for voluntary organizations in public health emergency services, so as to improve their capabilities in this field. It is well reflected in the existing policy documents. However, how to well organize some large-scale organizations, such as guilds with professional capabilities related to psychological support and grief counseling, is significant. Thus, more attention in this respect should be paid to policy design. They can also play an essential role in public health emergencies.

On comparing the service stages, the number of coded policy documents in the service delivery stages was similar to that in the service preparation stages. Among the service preparation stages coded in the policy documents, the codes of volunteer registration, recruitment, and training were the most. It may relate to the urgency and sudden characteristics of public health emergencies. The government deployed a variety of human and material resources to carry out the relevant prevention and control work. Besides the basic training and project planning, there are fewer requirements on the voluntary organization systems which need a long period to formulate. However, because of the temporary recruitment of volunteers, there was no systematic professional training for volunteers, which made the short-term training not guarantee effectiveness and reduced the value of volunteers. The government and voluntary organizations should sum up experience from this public health emergency to improve their defect, strengthen the construction of public health emergency prevention and control systems and working mechanisms, and promote their response capacity through specialization and systematic training, which will be beneficial for the better preparation for the future. Furthermore, in the service implementation stages, specific produced service contents, including public health emergency prevention and control, psychological counseling, popularization of medical knowledge and training, and assistance with daily life, were coded most in the policy documents. Tang et al. analyzed the current situation of emergency voluntary services in China during COVID-19 and found that the service contents were similar to the content mentioned in the policy, except for psychological counseling (29). The results of a multi-country comparative analysis of the impact of COVID-19 also showed that assistance to those suffering from psychological stress should not be overlooked during the COVID-19 recovery period (30). It indicates that there may still be a gap between policymaking and policy implementation, and environmental policies need to be improved to ensure the implementation of policies. Health service and monitoring, social network repairing, policy interpretation, referral service, grief counseling, and palliative and hospice care were less mentioned in the policy documents, which suggests the demand for more experts in these areas to serve as volunteers should be improved in the regular management period. A multinational survey that investigated voluntary services in palliative and hospice care during the COVID-19 pandemic demonstrated that volunteers were mostly prevented from supporting these services with the policy changes. The researchers also proposed that re-deployment plans are needed that take a more considered approach, using volunteers more flexibly to enhance care while ensuring safe working practices (31).

## Conclusion

Through the application of textual analysis, we analyzed the policy documents relating to volunteers participating in the prevention and control of public health emergencies in China and summed up the existing characteristics, advantages, and flaws. The policy has undergone three periods of evolution: incubation period, outbreak and continuous evolution period, and elimination recovery period. Issuing policy joint of various departments became the primary form, dominated by the National Health Committee with the Ministry of Civil Affairs and other departments as the local core, whose responsibilities matched best with the basic demands of humans' daily life and health. The internal structure of policy instruments was unbalanced and we expected it to be optimized to promote voluntary organization management, reinforce external resources, and close the gap between policymaking and policy implementation. The professional capacity of volunteers and the system of voluntary organizations needs to be improved, and the contents of prevention and control voluntary services should be enriched for preparedness for the future public health emergency.

## Data availability statement

The original contributions presented in the study are included in the article/Supplementary material, further inquiries can be directed to the corresponding author/s.

## Author contributions

HC, JW, XL, and YX: conceptualization. YX and XL: project administration and supervision. HC, XY, CL, and YZ: data curation, methodology, and software. HC and JW: writing—original draft. XL, JW, and YX: writing—review and editing. All authors contributed to the article and approved the submitted version.

## Funding

This study was supported by the Jiangsu Provincial Federation of Philosophy and Social Sciences Project (Grant No. 20SYB-114) and the Connotation Construction Project in Nanjing Medical University for Priority Academic of Nursing Science (Grant No. 2022-12).

## Conflict of interest

The authors declare that the research was conducted in the absence of any commercial or financial relationships that could be construed as a potential conflict of interest.

## Publisher's note

All claims expressed in this article are solely those of the authors and do not necessarily represent those of their affiliated organizations, or those of the publisher, the editors and the reviewers. Any product that may be evaluated in this article, or claim that may be made by its manufacturer, is not guaranteed or endorsed by the publisher.
